# Pregnant sows immunized with *Cryptosporidium parvum* significantly reduced infection in newborn piglets challenged with *C*. *parvum* but not with *C*. *hominis*

**DOI:** 10.1371/journal.pntd.0010690

**Published:** 2022-07-29

**Authors:** Abhineet Sheoran, Alison Carvalho, Ruby Pina Mimbela, Adam South, Samuel Major, Melanie Ginese, Donald Girouard, Saul Tzipori

**Affiliations:** Department of Infectious Disease and Global Health, Tufts University Cummings School of Veterinary Medicine, North Grafton, Massachusetts, United State of America; University of North Carolina at Chapel Hill, UNITED STATES

## Abstract

**Background:**

The piglet is the only model to investigate the immunogenic relationship between *Cryptosporidium hominis* and *C*. *parvum*, the species responsible for diarrhea in humans. Despite being indistinguishable antigenically, and high genetic homology between them, they are only moderately cross protective after an *active* infection.

**Methodology/Principal findings:**

Here we examined the degree of *passive* protection conferred to piglets suckling sows immunized during pregnancy with *C*. *parvum*. After birth suckling piglets were challenged orally with either *C*. *parvum* or *C*. *hominis* at age 5 days. Animals challenged with *C*. *parvum* had significant reduction of infection rate, while piglets challenged with *C*. *hominis* showed no reduction despite high *C*. *parvum* serum and colostrum IgG and IgA antibody.

**Conclusions/Significance:**

We add these data to earlier studies where we described that infection derived immunity provides partial cross-protection. Together, it appears that for full protection, vaccines against human cryptosporidiosis must contain antigenic elements derived from both species.

## Author summary

Cryptosporidiosis, a parasitic infection causing diarrhea and dehydration, emerged as a global enteric pathogen in the 1980s with the AIDS pandemic. *Cryptosporidium* infections have evolved to become a serious cause of morbidity and mortality in children under the age of 2 years in low to middle income countries. Given these developments, there is a critical need for an effective human vaccine. *C. hominis* and *C. parvum* are two *Cryptosporidium* species, with *C. hominis* being more common and more infectious than *C. parvum*. In this study, passive protection conferred to piglets suckling sows immunized with *C. parvum* during pregnancy was examined. Newborn piglets were separated into 4 groups: 2 groups from sows immunized with *C. parvum* and 2 control groups from unimmunized sows. One immunized group and one control group were infected with *C. parvum*, and the second immunized group and the second control group were infected with *C. hominis*. Newborn piglets were equally and fully protected against diarrheal disease regardless of whether the sow off which they fed was immunized. The results confirm that the dam’s colostrum and milk convey non-specific disease-preventing elements for full protection from disease, while passively acquired specific antibody reduces considerably the extent of infection.

## Introduction

Cryptosporidiosis had emerged as a global enteric pathogen in the 1980s with the AIDS pandemic, and more recently became recognized as a serious cause of morbidity and mortality in children under the age of 2 years in low to middle income countries (LMIC). In Uganda, we found that the 2-week case fatality rate in children was 13% [1]. In addition to acute diarrhea in healthy children, cryptosporidiosis may lead to persistent diarrhea in some (> 2 weeks), often leading to growth retardation, malnutrition, cognitive impairment, or death [2,3]. The Global Enteric Multicenter Study (GEMS) found *Cryptosporidium* to be 2^nd^ only to rotavirus as a cause of moderate to severe diarrhea (MSD) in children ≤ 2 years old [4–6]. More recently we have shown that 32% of infants with acute diarrhea with some respiratory symptoms had positive sputum for *C*. *hominis*, suggesting that aerosol transmission involving the respiratory tract also occurs, potentially adding to the maternal-child fecal-oral transmission cycle [1,7]. There is a critical need for an effective human vaccine against this infection to protect children in LMIC.

Two enteric *Cryptosporidium* species, *C*. *hominis* and *C*. *parvum* cause human disease, with *C*. *hominis* being by far (>75%) more common and more pathogenic than *C*. *parvum*. In 243 Ugandan children <3 years, we have shown that 1/3 had chronic diarrhea due to cryptosporidiosis, of whom 74% excreted *C*. *hominis*, while only 18% had *C*. *parvum*, 4% a mixture of both, and 4% had *C*. *meleagridis*, an avian species [8].

The newborn gnotobiotic (GB) piglet is the only mammal (other than human volunteers) that develops diarrhea following challenge with *C*. *hominis*. Using GB piglets, we have already shown that primary infection with *Cryptosporidium* protects piglets against secondary challenge providing the first evidence that parasite antigens induce complete homologous but only partial heterologous protective immunity [9,10].

In this communication, we extend these observations to demonstrate a similar pattern with passively acquired specific antibody. Whereas piglets suckling sows immunized with *C*. *parvum* during pregnancy significantly resisted homologous parasite challenge, piglets challenged with *C*. *hominis* excreted oocysts in their feces the same as piglets suckling unimmunized sows.

## Materials and methods

### Ethics statement

All experiments were performed in strict accordance with the recommendations set forth by the National Institutes of Health Guide for the Care and Use of Laboratory Animals (8th Edition). Protocols were approved by the Institutional Animal Care and Use Committee at Tufts University Cummings School of Veterinary Medicine (Animal Welfare Assurance Number D16-00572 [A4059-01]). All efforts were made to minimize animal suffering and distress over the course of studies performed.

### *Cryptosporidium* isolates and preparation of sonicated antigens

*C*. *parvum* oocysts (Iowa strain) were purchased (Bunch Grass Farm, Drury, ID). As a large number of *Cryptosporidium* oocysts were required, it was not possible to generate them in house. *C*. *hominis* oocysts (TU502 strain) were originally isolated from Ugandan children with diarrhea [11,12] and maintained through continuous propagation in gnotobiotic piglets for over a decade and half. *C*. *hominis* oocysts for challenge experiments were purified from piglet feces and genotyped as described [11,13]. The oocyst excystation rate was estimated as a measure of oocyst viability.

For preparation of *Cryptosporidium* antigens, 10^9^ *C*. *parvum* or *C*. *hominis* oocysts were bleached in 1 ml 10% bleach over ice for 7 min. Oocysts were centrifuged at 15,000 × g in a microcentrifuge for 1 min and washed 3 times with PBS by resuspending the oocyst pellet each time 1 ml sterile PBS and centrifuging it. The pellet was frozen/thawed 7 times (freezing in liquid nitrogen and thawing in a 37°C water bath, 2 mins each) in PBS to break open unexcysted oocysts and sporozoites, and sonicated for 20 min over ice. Protein concentration was determined and antigens were stored at -20°C.

### Passive protection experiments in conventional suckling piglets

Two pregnant sows (#4 and #6; Table 1), approximately 45 days into gestation, were immunized 6 times with *C*. *parvum*, beginning with two oral immunizations of 10^9^ live *C*. *parvum* oocysts, given a week apart. This was followed up with 4 parenteral immunizations (intramuscular) at 14 day intervals with sonicated sporozoite antigens of *C*. *parvum*. They were injected intramuscularly with 2 mg per immunization admixed with 300 μg of CpG (ODN2395, Invivogen) and Alhydrogel (Invivogen) adjuvants to help induce both T helper 1 (Th1) and Th2 responses. Two unimmunized pregnant sows (#2 and #7; Table 1) were included as control.

After birth, piglets suckling sows #2 and #4 were each challenged orally on day 5 with *C*. *parvum*, while piglets of sows #6 and #7 were similarly challenged orally with *C*. *hominis* (Table 1). All piglets suckled their respective dam. Piglets were bled before the challenge to determine serum antibody titers, monitored daily for diarrhea and onset, level and duration of oocyst excretion in feces for 17 days after parasite challenge. Our efforts to collect fecal samples from individual piglets was unproductive as suckling piglets are routinely cleaned by their dam. Therefore, rectal swabs were instead collected daily, suspended in 100 μl of sterile water, and aliquots thereof were spread onto microscopic slides, stained with modified acid fast, and oocysts were enumerated microscopically under 100 × magnification [14].

### Enzyme linked immunosorbent assay

*C*. *parvum*-specific antibody titers in serum or colostrum were evaluated using indirect enzyme linked immunosorbent assay (ELISA). Polystyrene high binding 96-well ELISA plates (Greiner Bio One) were coated with 100 μl/well of sonicated *C*. *parvum* antigens in phosphate buffered saline (4 μg/ml) and incubated overnight at 4°C. The plates were washed three times in Tris-Buffered Saline + 0.1% Tween-20 (TBS-T) after each incubation step. The plates were blocked with 100 μl/well of 2% non-fat milk powder in TBS-T and incubated for 1 h at 37°C. 100 μl/well of TBS-T was added to the entire plate. Serum or colostrum samples were added in duplicate (100 μl/well) diluted two-fold serial along the rows. A serum from an unimmunized pig was used as a negative control. The plates were incubated at 37°C for 1 h. 100 μl/well of horseradish peroxidase (HRP) conjugated goat anti-pig IgG (1/10,000 in TBS-T) or IgA (1/4000 in TBS-T) (Bethyl Laboratories) antibody was added to the entire plate. The plate was incubated at 37°C for 1 h. Substrate solution (10 mg *O*-Phenylenediamine, 25 μl 30% hydrogen peroxide, and 15 ml 0.1 M citrate phosphate buffer, pH 5.0) was added 100 μl/well and the plates developed for 10 minutes. The reaction was stopped by adding 50 μl/well of 2 M H_2_SO_4_ and the absorbance read at 490 nm. The titer for each serum or colostrum sample was the reciprocal of the last dilution that gave mean optical density (OD) value twice that of the mean OD value of the negative control at the same dilution.

## Statistical analysis

Assumptions of normality and homogeneity of variance for all data were checked using the Shapiro-Wilks and Levene’s test, respectively. The non-parametric Mann-Whitney test was used to determine if there were differences in antibody titers between our treatments in animals challenged with *C*. *hominis* or *C*. *parvum*. The Mann-Whitney test was also used to characterize putative differences in the number of oocysts shed between the piglets of the control and immunized sows. All analyses were done using SPSS version 25.

## Results

### Specific *C*. *parvum* IgG and IgA antibody in serum and colostrum of the sows

The two control sows showed variably low or absence of preexisting serum (#2) or colostrum IgA (#7) antibody. Immunized sows #4 and #6 with *C*. *parvum* antigens in contrast generated high serum and colostrum IgA and IgG ELISA titers against *C*. *parvum* (Table 1).

**Table 1:** Summary of immunization of pregnant sows, their ELISA antibody titers, the number of piglets born to each, and the dose and parasite species the piglets were challenged with.

Sow #	Immunized /control	Serum* IgG/IgA	Colostrum* IgG/IgA	Piglets born	Challenged (10^6^ oocysts)
2	Control	0/16	0/16	9	*C*. *parvum*
4	*C*. *parvum*	512/64	512/128	10	*C*. *parvum*
6	*C*. *parvum*	128/64	512/256	10	*C*. *hominis*
7	Control	32/0	64/0	6	*C*. *hominis*

*Reciprocal ELISA titers x 1000

### Passive transfer of *C*. *parvum* colostrum antibody from immunized sows to piglets

**Fig 1:**
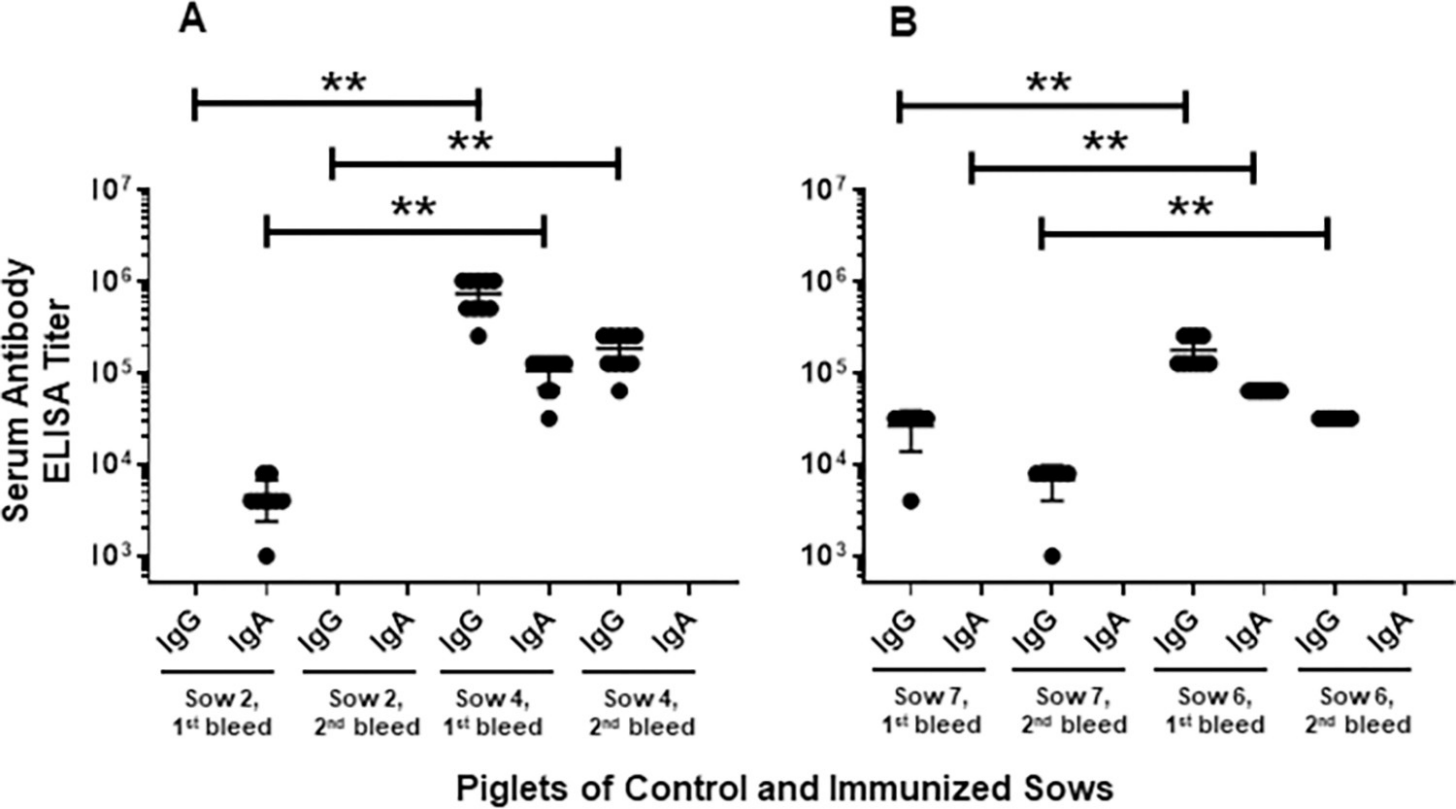
Serum IgG and IgA levels (reciprocal ELISA titers) of piglets before the parasite challenge (1^st^ bleed) and at the end of the experiment (2^nd^ bleed) against *Cryptosporidium parvum*. (A) Serum IgG and IgA levels of piglets of the control (sow 2) and immunized (sow 4) sows that were challenged with *C*. *parvum*. (B) Serum IgG and IgA levels of piglets of the control (sow 7) and immunized (sow 6) sows that were challenged with *C*. *hominis*. **p <0.0005.

Piglets suckling control sow #2 had undetectable serum IgG and low levels of serum IgA (titers ranged 1,000–8,000; mean, 4,556) against *C*. *parvum* before challenge with *C*. *parvum* oocysts, which became undetectable at the end of the experiment (Fig 1A).

Before challenges, piglets suckling immunized sow #4 had high levels of serum IgG (titers ranged 256,000–1,024,000; mean, 742,400) and IgA (titers ranged 32,000–128,000; mean, 105,600) levels against *C*. *parvum* (1^st^ bleeds, Fig 1A). These serum IgG and IgA levels of piglets of sow #4 were significantly higher than the piglets suckling control sow #2 (*p* <0.0005).

Piglet sera were collected from all 35 animals at the end of the experiment on day 17 after parasite challenge. Piglets suckling immunized sow #4 had strong serum IgG levels (titers ranged 64,000–256,000; mean, 185,600) at the end of the experiment (2^nd^ bleeds, Fig 1A), which were significantly higher than the piglets of control sow #2 (*p* <0.0005). However, neither immunized nor control sow piglets had detectable serum IgA against *C*. *parvum* by the end of the experiment.

Piglets of control sow #7 had undetectable serum IgA and low levels of serum IgG before challenge with *C*. *hominis* oocysts (titers ranged 4,000–32,000; mean, 26,400) and at the end of the experiment (titers ranged 1,000–8,000; mean, 6,833) (Fig 1B). Piglets suckling sow #6 had high levels of serum IgG (titers ranged 128,000–256,000; mean, 179,200) and IgA (a titer of 64,000 for all piglets) levels against *C*. *parvum* (1^st^ bleeds, Fig 1B), which were significantly higher than those of the piglets of the control sow #7 (*p* <0.0005). Piglets suckling sow #6 did not have detectable serum IgA against *C*. *parvum* by the end of the experiment but had moderate levels of IgG (a titer of 32,000 for all piglets), which were significantly higher than those of the control sow #7 piglets (*p* <0.0005) (Fig 1B).

### Serum *C*. *parvum* antibody reduces infection in piglets challenged with *C*. *parvum*

**Fig 2:**
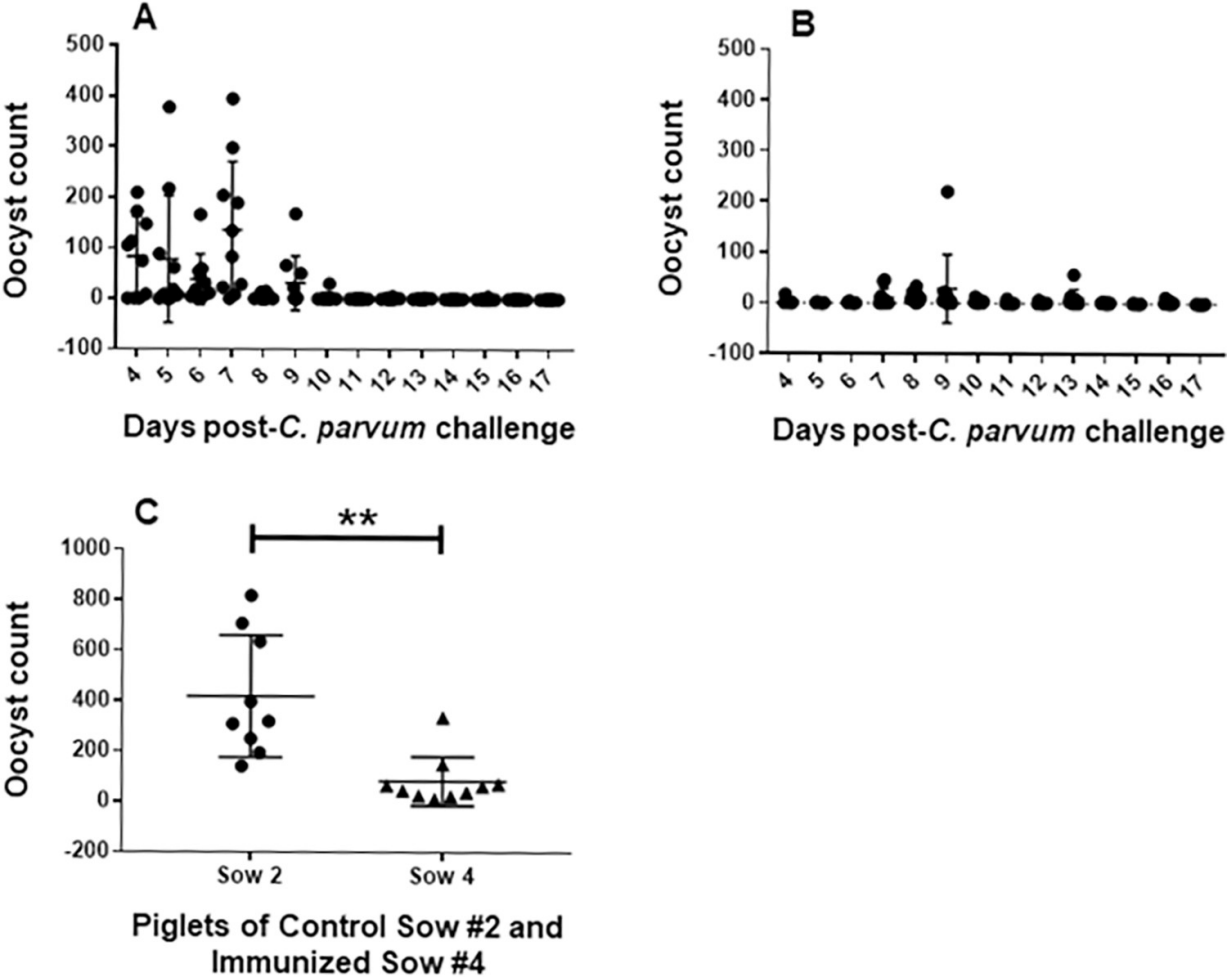
Daily oocyst excretion pattern following infection of the piglets with *Cryptosporidium parvum*. A) piglets of the control sow #2, and (B) immunized sow #4. (C) total oocyst excretion of each piglet of control and immunized sows over 17 days post-infection **p <0.0005.

Piglets suckling control and immunized sows began to excrete oocysts in their feces 3–4 days post-infection (Fig 2A and 2B). None of the piglets of control or immunized sows developed diarrhea during the 17 day post-infection period. All piglets of control sow #2 excreted oocysts on days 6 and 7 after challenge, and 6 of the 9 piglets on day 9. All piglets of unimmunized sows shed oocysts on intermittent days after day 9 except for 2 piglets which did not excrete oocysts after day 9 (Fig 2A). Unlike control sow #2 piglets, the majority of the piglets of the immunized sow #4 continued to excrete oocysts for longer (days 13, 14 and 16 post-infection). Overall, the mean of total oocyst excretion of each piglet over 17 days post-infection was significantly higher in the control sow #2 piglets than the immunized sow #4 piglets (*p* <0.0005, Fig 2C). There does not seem to be a correlation between antibody levels and extent of protection in piglets of the immunized sows, as piglets with high antibody titers were not protected any more than the piglets with lower antibody titers. This may well be due to the amount of secretory IgA and IgG the immunized sow had provided in the colostrum and milk during the course of infection which protected the gut (S1– S4 Tables).

### Serum *C*. *parvum* antibody has no benefit to piglets challenged with *C*. *hominis*

**Fig 3:**
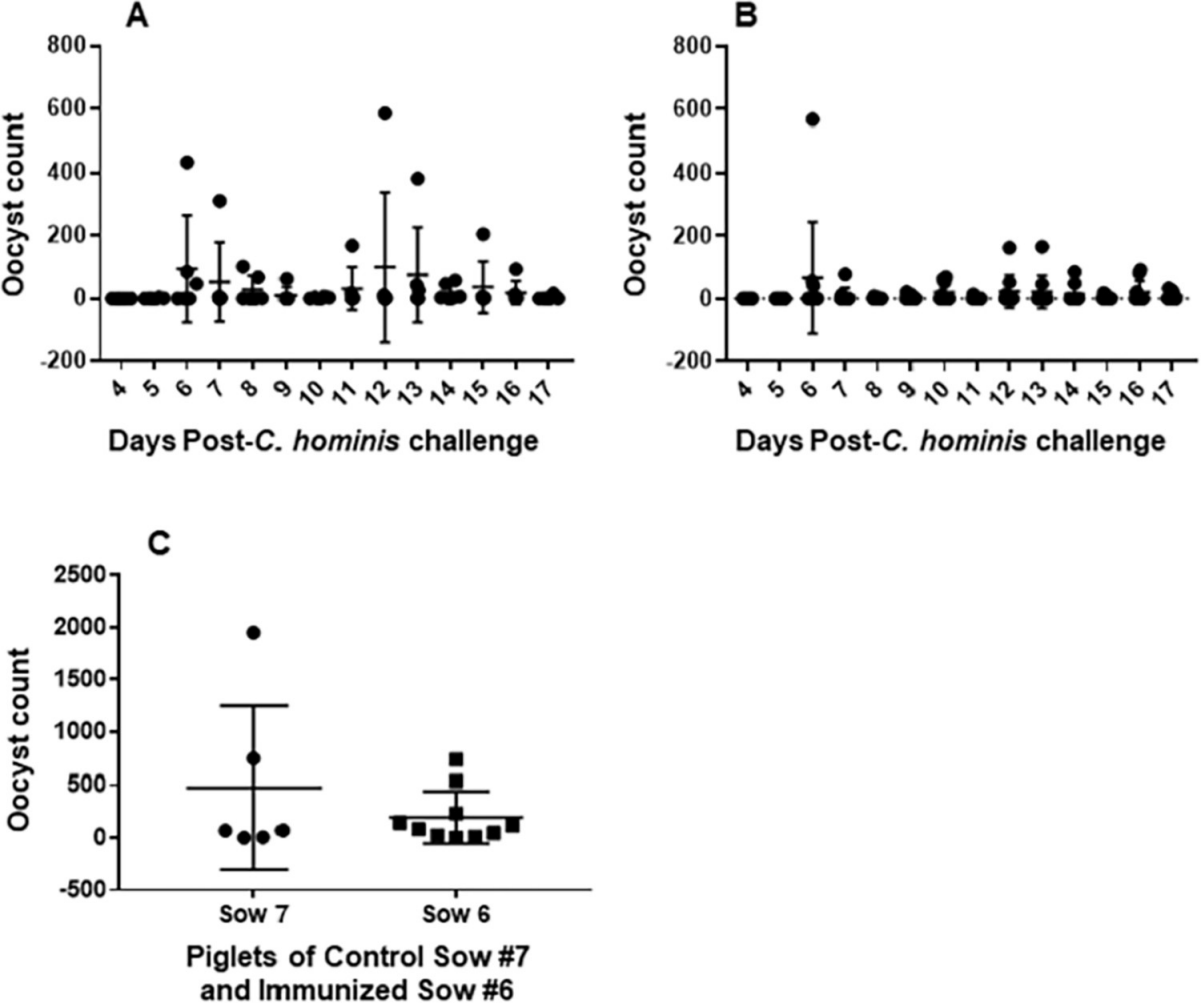
Daily oocyst excretion pattern following infection of the piglets with *Cryptosporidium hominis*. (A) piglets of the control sow #7, and (B) immunized sow #6. (C) total oocyst excretion of each piglet of control and immunized sows over 17 days post-infection.

Some piglets suckling control sow #7 and immunized sow #6 started to excrete oocysts on days 5 and 6 post-infection, respectively (Fig 3). A single animal suckling control sow #7 did not shed oocysts at all, 3 shed for 10–12 days and 2 shed for 3–6 days (Fig 3A). Six of the 10 piglets suckling immunized sow #6 shed oocysts for 1–4 days and 4 shed for 6–9 days (Fig 3B). Overall, the mean of the total oocyst excretion of each piglet over 17 days post-infection did not differ significantly between the control and immunized sow piglets.

## Discussion

*C*. *hominis* are known to infect and cause serious disease in humans only, whereas *C*. *parvum* is found commonly in all mammals, and are known to cause acute diarrheal disease in humans and in newborn ruminants. While cryptosporidiosis attributed to these two species occurs worldwide in the human population of all ages, their impact is consequential largely in children under the age of 2 years living in LMIC where vaccines are urgently needed.

It is curious that despite the lack of clearly identifiable antigenic differences between *C*. *parvum* and *C*. *hominis* and the very high degree of genomic homology between them [11,12], profound differences are known to exist with their host predilection [13,14]. While *C*. *hominis* is human-restricted, we demonstrated over two decades ago that *C*. *hominis* is amenable to infecting newborn piglets in which it induces a serious acute diarrhea as seen in children. Piglets, which are also susceptible to *C*. *parvum*, makes the pig, a large animal, uniquely suitable to conduct comparative studies between the two parasite species [14]. This precluded the use of a higher number of animals per experiment, as is possible when using rodent models. In addition, unlike humans and mice, most domestic animals, including swine, are born agammaglobulinemic and receive all their maternal systemic IgG and mucosal secretory IgA antibody via the colostrum after birth.

Cross-infection studies performed in GB piglets have shown poor to moderate *active* cross-protection between them [9,10]. This indicates that infection or immunization would likely only guarantee full protection against the homologous species, and, at best, provide only partial protection against the heterologous species. This has serious consequences for the design of future vaccines against this parasite, taking into consideration that vaccines designed for LMIC in whom *C*. *hominis* is found in ~75% of cases should be the main focus of vaccine development [4,6].

The outcome of the current studies, which focus on the role of *passive* protection, showed that newborn piglets suckling immunized, or control sows were equally and fully protected against diarrheal disease, as none of the 35 challenged animals with high infectious doses of either *C*. *parvum* or *C*. *hominis* experienced symptoms of disease regardless of the level of specific antibody measured in the serum, colostrum and milk. We did not attempt to immunize pregnant sows with *C*. *hominis* because logistically it would not have been possible to generate sufficient oocysts in gnotobiotic piglets for repeated immunization of pregnant sows. Nonetheless, our results indicate, yet again, the probable presence of non-specific disease-preventing elements in the dam’s colostrum and milk, coming from either a mother or a sow, since human infants are also protected from disease for as long as they are breastfed over the first 6 months of life. Thereafter children become susceptible to serious disease when infected after weaning [1–6]. Gut microbiome may also have contributed to protection of suckling piglets when comparing the outcome of infections between sows’ milk fed suckling piglets and age-matched gnotobiotic piglets fed a sterile infant milk diet. Compared to older animals, newborn piglets develop antibody after infection at a slower rate than ≥4 weeks, and it is likely that, at the time of euthanasia, the systemic Ab levels were below a detectable level in the control sow #2 (Fig 1A). The presence of systemic Abs in piglets suckling immunized sows is of maternal origin, absorbed from the colostrum immediately after birth.

The levels of anti *C*. *parvum* antibody in serum and colostrum provided by sow #4 compared with unimmunized sow #2, however, had a significant (*p*<0005) impact on the total amount of oocyst excretion measured among piglets suckling immune vs. unimmune dams after challenge with *C*. *parvum*. In contrast, despite the high anti-*C*. *parvum* antibody in the serum and colostrum of sow #6, her suckling piglets were as susceptible to *C*. *hominis* infection in terms of oocyst excretion as those suckling the control sow #7 (Fig 3). Earlier studies have also shown that passively transferred antibodies to newborns through the colostrum induced by immunizing ruminant (cows, sheep, goats) dams with *C*. *parvum* [15] or its recombinant antigen [16] or a DNA vaccine [17] provided incomplete protection against *C*. *parvum* infection [15–17].

In conclusion, despite the limited number of pregnant sows used in this study, our results provide strong evidence that there is only partial cross-protection among the two parasite species that infect humans. This is consistent with observations we reported earlier with active cross-protection. Yet, none of the 35 challenged suckling piglets, although they became infected, developed diarrhea, presumably due to non-specific protective elements in the milk, somewhat enhanced by the presence of specific homologous antibody. This is evident since differences were observed in the level and duration of oocyst excretion in the feces, showing that piglets suckling dams immunized with *C*. *parvum* experienced a significant reduction in the infection rate when challenged with homologous *C*. *parvum* compared to those challenged with the heterologous *C*. *hominis*. Together these observations provide some evidence that passive, systemic and milk antibodies, as presumably with children, while providing protection against disease they offer only limited protection against infection with a homologous, and minimal to none against the heterologous parasite species.

## Supporting information

S1 Table Daily Cryptosporidium parvum oocyst excretion pattern following infection of the piglets of control sow #2 with C. parvum, and serum antibody titers on day of the infection (first bleed) and at euthanasia (second bleed). (XLSX)

S2 Table Daily Cryptosporidium parvum oocyst excretion pattern following infection of the piglets of sow #4 immunized with C. parvum, and serum antibody titers on day of the infection (first bleed) and at euthanasia (second bleed). (XLSX)

S3 Table Daily Cryptosporidium hominis oocyst excretion pattern following infection of the piglets of control sow #7 with C. parvum, and serum antibody titers on day of the infection (first bleed) and at euthanasia (second bleed). (XLSX)

S4 Table Daily Cryptosporidium hominis oocyst excretion pattern following infection of the piglets of sow #6 immunized with C. parvum, and serum antibody titers on day of the infection (first bleed) and at euthanasia (second bleed). (XLSX)
